# The impact of swaddling upon breastfeeding: A critical review

**DOI:** 10.1002/ajhb.23878

**Published:** 2023-02-14

**Authors:** Allison Dixley, Helen L. Ball

**Affiliations:** ^1^ Durham Infancy & Sleep Centre, Department of Anthropology Durham University Durham UK

## Abstract

**Introduction:**

Many parents swaddle their infants to promote sleep and reduce night‐waking, however lack of definitive evidence about the pros and cons of swaddling when breastfeeding hinders postnatal recommendations regarding this infant care practice. This review critically examines research conducted on the impact of swaddling upon breastfeeding.

**Methods:**

Only two recent studies on swaddling outcomes have reported infant feed‐type, therefore the purpose of this paper is to consider the known effects of swaddling on breastfeeding babies and their mothers. We interpret the existing literature on swaddling in terms of impact on breastfeeding physiology and behaviour during the immediate post‐natal period, and as infancy progresses.

**Results:**

Infants swaddled immediately after birth show a delay in initial breastfeeding, less successful suckling at the breast, reduced intake of breastmilk and greater weight loss compared to un‐swaddled babies. Swaddling visually obscures feeding cues and reduces crying, thereby eliminating two key feeding prompts typically used by parents/carers.

**Conclusions:**

As swaddled babies cry less, and are fed less frequently than un‐swaddled babies some clinical trials position swaddling as a ‘novel weight regulation tool’ to combat obesity. However, in the case of breastfed babies, by reducing feed frequency swaddling may impede maternal milk production and thereby infant growth.

## 1 INTRODUCTION

Despite being a requirement for infant survival through most of our evolutionary and historical past, the emphasis placed on breastfeeding within contemporary public health infant feeding policy is not carried through to other areas of infant care, the research and recommendations regarding which often ignore any impact on breastfeeding outcomes. Research examining the effects of infant swaddling generally overlooks how swaddling might affect breastfeeding outcomes.

The influence of swaddling on breastfed babies or breastfeeding outcomes has not been critically reviewed. In a recent systematic review of swaddling on sleep and arousal outcomes focussing on studies post‐2007 (Dixley & Ball, 2022), we found that only two swaddling studies conducted in a 15‐year period reported on feeding method for the infants studied (Öztürk & Bayik, 2019; Richardson et al., 2010). Given the lack of attention to breastfed babies in recent studies of swaddling, the fact that many parents swaddle their infants to promote sleep, and the observation that cultures in which infant sleep interventions are popular also exhibit poor rates of sustained breastfeeding (Ball & Taylor, 2020), we undertook this review to critically examine the known and potential consequences of swaddling for breastfeeding, and for breastfed babies.

A systematic review of swaddling studies conducted prior to 2007 reported no relationship between swaddling and breastfeeding (van Sleuwen et al., 2007), however it was notable that every randomized trial examined in that review involved swaddling that took place in the context of other practices that impeded instinctual interaction between infants and their mothers, for instance, the use of incubators or pacifiers. Here we review studies of swaddling from 2007 onwards identified via a previous systematic review (Dixley & Ball, 2022) to specifically examine the contexts in which swaddling studies were conducted, and their potential impacts on breastfeeding, as well as any reported outcomes.

### 1.1 Breastfeeding initiation in the immediate postnatal period

Two studies published in 2007 reported seemingly contradictory findings on the effect of swaddling in the immediate postnatal period. In a study of Russian maternity home routines, Bystrova et al. (2007) observed that infants swaddled in the first few days after birth experienced a delay in initial breastfeeding, less successful suckling at the breast, reduced intake of breastmilk and greater weight loss than those who were not swaddled. As swaddling was not a randomized intervention in this study it is impossible to ascertain why a particular baby may or may not have been swaddled, and whether infant attributes affecting feeding outcomes may have also prompted swaddling (e.g. prematurity).

In contrast, in a small randomized trial, Moore and Anderson (2007) found infants who suckled competently during their first 2 h of life did so regardless of whether they experienced skin‐to‐skin contact with their mother or were swaddled. At face value this suggests that swaddling may have little impact on breastfeeding initiation. However, these findings must be considered with caution given the small sample size (*n* = 20), the mothers' robust motivation to breastfeed, and the focus on infants with good suckling competence.

Surprisingly an often‐overlooked participant in the debate over breastfeeding and swaddling is the infant themselves. In reference to the immediate postnatal period, Tow and Vallone (2009) stress that “the competency of the infant at birth is a biological imperative” for breastfeeding establishment (p.626). After an unmedicated labour and delivery, unswaddled newborn human infants placed onto their mothers' stomachs will crawl and squirm towards the nipple, guided by smell, and then suckle without assistance (Nissen et al., 1995; Varendi et al., 1994; Varendi & Porter, 2001). The same infants will perform an instinctive pattern of ‘kneading’ hand movements that have been shown to increase maternal oxytocin levels and facilitate milk let‐down, a behavior which continues through infancy (Matthiesen et al., 2001). Although it is well documented that physical separation of babies from their mothers immediately post birth (e.g. by use of bassinets and incubators) impedes adaptive behaviors such as the breast crawl and kneading hand movements (Henderson, 2011; Bramson et al., 2010.), it is often overlooked that swaddling is also a form of separation that likewise impedes the expression of adaptive behaviors. In forming a physical barrier between mother and infant, swaddling prevents the continuity and postnatal effectiveness of the maternal body as the expected environment for her neonate.

As swaddling prevents skin to skin contact and the expression of infant instinctive behaviors it therefore has the capacity to undermine feeding as a relational, inter‐embodied practice (Tomori et al., 2016), placing swaddled infants at a disadvantage in comparison to their non‐swaddled counterparts. Infants that experience skin‐to‐skin contact immediately post‐birth are more successful at establishing breastfeeding and breastfeed for more months than those who do not experience skin‐to‐skin contact (Anderson et al., 2003; Cadwell et al., 2018; Singh et al., 2017; Tosun Guleroglu et al., 2020; Vila‐Candel et al., 2018). Lupton describes this dynamic as “skinship”, the relational state created by close physical proximity, touch and intimacy (Lupton, 2013 p.40). These factors form an adaptive and bidirectional complex recognized in those cultures (Ball & L., 2019; Bonamy, 2016; McKenna & Gettler, 2016a; McKenna & Gettler, 2016b) where infants are attached to their mother's body, in continuous proximity to the smell of milk, and always ready to be fed (Hewlett et al., 1998; Hewlett & Lamb, 2002; Morelli et al., 1992; Richman et al., 1992; Sansone, 2004). Such instinctive behavior of the maternal–infant complex (or mother‐infant nexus) is considered unusual and even controversial under the Western biomedical model (see Gowland & Halcrow, 2020; Van Esterik, 2017).

### 1.2 Separation and biobehavioural consequences in the early postnatal period

Arising from a marriage of capitalism and industrialization, Western biomedicine has emphasized the separation of bodies to ensure ‘safety’ (Ball, 2002; Ball, 2003; Alexeyeff 2013; Ball et al. 2019) with a goal of minimal and eventually no night‐time breastfeeding (Tomori, 2014; Tomori et al. 2016). As infants are naturally inclined to seek more parental resources than it is in the mother's interest to provide (Stuebe & Tully, 2019; Trivers, 1974), swaddling may be viewed as a maternal strategy to balance the trade‐off between her infants' care needs and her personal wishes for uninterrupted sleep, bodily autonomy and individuality (Tully & Ball, 2018).

Physical separation is associated with decreased breastfeeding (McKenna et al., 1997; Hauck & Kemp, 1998; Ball et al., 2006; McKenna et al. 2007). Even separation via the wall of a hospital bassinette placed next to the maternal bed has been shown to exert physiological and psychological influences on the behavioral and biological relationship between infant and mother (Ball, 2008; Ball et al., 2006). Nonetheless, the swaddling of babies has been practiced for a long time among some non‐Western populations. The Wari of Amazonia for instance, adopt the concept of the ‘external womb’ whereby after biological birth, the infant remains socially unborn during which time they are swaddled and attached to their mother with a piece of material contributed by the father (Conklin & Morgan, 1996; Lancy, 2014). In Romanian culture, newborns are reportedly starved for the first 3 days of life, swaddled tightly in their parents' clothing and tied up with a chain to make them “strong as iron” (Borbely, 1999:27, Dervin, 2008:220). Likewise, many contemporary Turkish infants are swaddled at some point during the first year of life (Çağlayan et al., 1991; Kutlu et al., 1992; Akman et al., 2007; Okka et al., 2016) until they “seem strong and healthy” (Delaney, 2000:131). However, few ethnographic reports of swaddling also discuss infant feeding practices.

In Thailand, where ‘moderately snug’ swaddling is widely practiced and supported alongside breastfeeding and rooming‐in, a breastfeeding initiation rate of 91.2% within 1 h of delivery, and an average breastfeeding duration of 19 months was reported for full‐term babies (White et al., 2012). This study suggests that along with a strong intent to breastfeed, a supportive breastfeeding environment may ameliorate any potential effects that swaddling might have on breastfeeding initiation; in this case swaddling was only implemented after an initial period of skin‐to‐skin contact and breastfeeding initiation in over 90% of cases.

An emerging hypothesis in the field of infant nutrition considers whether parenting style influences responsiveness to hunger cues or whether responsiveness is simply part of distinct parenting beliefs or ‘parental ethnotheories’ (Harkness & Super, 2006; Little et al., 2018). In exploring this hypothesis, Little and colleagues (Little et al., 2018) found that mother–infant physical contact, reflected in parenting practices such as bedsharing or sling‐use, increased maternal responsiveness to infant hunger cues in comparison with visual cues only (i.e. without physical contact). This finding remained even after parental beliefs about responsiveness were controlled for. The authors concluded that there is “something special about direct physical contact that facilitates infant‐led motivations for feeding above and beyond just having the infant in proximity”.

Multiple researchers have recommended a responsive approach as the optimal feeding strategy (Woolridge, 1995; Brown & Arnott, 2014; Shloim et al., 2017). Responsive feeding involves the caregiver initiating feeding upon noticing the infant's display of early hunger cues and then terminating feeding when the infant shows signs of satiety (American Academy of Pediatrics, 2017). Early hunger cues include mouthing hands or objects, making sucking noises or motions, clenching fingers or fists over the torso, flexing arms and legs, or engaging the rooting reflex. Crying, on the other hand, is considered a ‘late’ hunger cue (Hodges et al., 2008; American Academy of Pediatrics, 2017). By limiting infant movements swaddling obscures (and supresses) early infant feeding cues and provides a proximal barrier to mother‐infant interaction, separating carers and babies visually as well as physically.

Recent research underscores a strong association between breastfeeding outcomes and proximity of the infant to the mother (Bailey et al., 2020). In this regard, swaddling might be considered a double‐edged sword: the visual obscuring or suppression of feeding cues means that crying is one of the few behaviors a swaddled infant can adopt to communicate their hunger. In Western industrialized societies, crying is the most commonly reported hunger cue used by mothers to initiate feeding (Gross et al., 2016). However, as discussed below, swaddling suppresses infant crying overall, and as swaddled infants cannot engage in physically active feeding behavior, swaddling appears to cultivate an infant care environment more compatible with bottle‐feeding. As breast‐fed infants commonly use their hands to touch and knead the breast during feeding, lactation specialists maintain that breastfed infants should not have their hands anchored away from the breast by swaddling (Cadwell, 2007). Consequently, swaddling has been criticized by midwives who argue it “accentuates the discontinuity of postnatal transition” and “assumes that the continuity of maternal nutrition ends at birth” (Colson, 2002 p.61).

Responsive feeding is also invoked in the concept of ‘shared feeding responsibility’, recently promoted to prevent obesity from as early as infancy. This concept positions parents in the role of provider of healthy food whilst the child's role is to decide how much to eat based on their hunger and satiety cues (Paul et al., 2018). It can be argued that swaddling undermines both the mother's and the infant's role in the shared feeding exchange. Some pediatricians advocate swaddling prior to feeding (Karp, 2018), however, the barrier of swaddling prohibits skin‐to‐skin contact, thus undermining mothers' regulatory abilities. For breastfeeding mothers, swaddling negatively interferes with the ability to adopt optimum positioning (Charlmers, 2005). One lactation specialist observed that swaddling encourages a mother to “cradle her cueless, clueless baby supine in her arms, being sure to support the head, while trying to point a nipple into his mouth” (Genna, 2017 p.127). This positioning, she highlights, is not conducive to successful breastfeeding.

To breastfeed successfully, infants typically need to actively coordinate sucking, swallowing, breathing, and cardiorespiratory patterns (Sakalidis & Geddes, 2016). For infants, tight swaddling may impede this coordination, hindering their capacity to mold their body to their mother's, to nestle, and to actively move in order to participate in the activity of feeding (as opposed to the passivity of being fed). The continuous sensory load of swaddling may be particularly problematic for infants described as highly sensitive to touch; the gag reflex of such infants can be triggered by pressure applied to non‐oral body parts, such as the arm or shoulder (Scarborough et al., 2006), locations typically stimulated via swaddling.

### 1.3 Swaddling and feed frequency during late postnatal period

In addition to feed technique there is a growing appreciation of the fundamental role of feed frequency in modulating and sustaining human milk supply. The process of lactation involves two maternal hormones: prolactin triggers milk‐production while oxytocin stimulates the release or ‘let down’ of the milk. A variety of stimuli can trigger the production of these hormones, including the sight, smell, sound and feel of an infant (World Health Organization, 2009). A hormonal surge is experienced with every feed or attempted feed, and around 45 min after feeding, the surge declines. Repeated feeding maintains circulating blood prolactin levels and protects a robust milk supply (Tennekoon et al., 1994; Woolridge, 1995; Neville, 2001). Consequently, authorities such as the Centres for Disease Control and Prevention maintain that breastfed infants will feed eight to twelve times every 24 h (CDCP, 2022). Swaddled infants, however, have been found to feed no more than six or seven times in a 24‐h period (Franco et al., 2005). While having her baby sleep more might be perceived as a benefit to the mother, the breastfed infant would appear to be disadvantaged.

One potential reason for this discrepancy is that swaddling undermines or mediates the biobehavioural processes associated with hunger. Firstly, the increased cortisol production associated with mother‐baby separation reduces infant appetite (McKenna, 2016). Swaddling is a form of physical separation given that it prohibits skin‐to‐skin contact. Scholars have reported that weight gain correlates positively with the amount of daily touch infants receive, leading some to hypothesize that physical touch may make the absorption of calories more efficient by speeding up digestion and reducing agitation‐related energy expenditure (Field, 1995 as cited in McKenna, 2016; Juneau et al., 2015). How swaddling affects metabolic efficiency should therefore be investigated.

As well as potentially suppressing behavioral systems associated with hunger, it is well established that swaddling reduces infant sleep arousal. Swaddled infants wake less frequently than when un‐swaddled (Franco et al., 2005). This is problematic for all infants but particularly breastfed infants, as a reduction in feeds affects the bio‐behavioral synchrony of the mother‐infant dyad, potentially reducing the mother's milk supply. The phenomena of ‘arm cycling’ gives a possible clue to one of the mechanisms at play. When infants progress from deep to light sleep, their arms begin to wave, or ‘cycle’ (Gerard et al., 2002; Richardson et al., 2010). Scholars argue that this arm movement serves as a cue to help wake the infant for feeding (Colson et al., 2003; Fauntleroy, 2012). Therefore, when the arms are bound by swaddling, the infant may cycle back into quiet sleep and miss a feeding opportunity. This observation demonstrates how variations in sleep ecology may have substantial consequences for the functioning of maternal lactation biology (Ball, 2008; Ball, 2019). Infants are efficient at suckling and swallowing during both sleep and awake states (Colson, 2010), suggesting that a considerable part of milk intake may occur during sleep for those infants who are permitted unhindered access to the breast (Rosin, 2019).

In contrast to the above perspective, Van Sleuwen and L'Hoir (2007) found that swaddling was associated with a reduction in ‘excessive crying’ and speculated that this may be useful in preventing overweight and obesity, “as parents of excessively crying infants have the tendency to offer extra feeds” (van Sleuwen & L'Hoir, 2007 p.3). Van Sleuwen and L'Hoir cite lengthened sleep as another virtue of swaddling in the ‘war against obesity’, given the possible link between obesity and short sleep duration (Taheri, 2006).

The intriguing prospect that infant sleep duration may be a modifiable risk factor for obesity has inspired several US‐based clinical trials (Gillman et al., 2008; Redsell et al., 2011; Anzman‐Frasca et al., 2013; Gross et al., 2016; Savage et al., 2016; Paul et al., 2018; Lavner et al., 2019; Harris et al., 2020). Among them were the Healthy Beginnings Trial (Wen et al., 2012), the Intervention Nurses Start Infants Growing on Healthy Trajectories (INSIGHT) (Paul et al., 2014) and NOURISH‐RCT (Daniels et al., 2012). Although exact mode of feeding is often unclear, a common feature of each trial was the introduction of swaddling. Grounded in developmental theory on parenting sensitivities, the conceptual framework employed in these trials maintains that by promoting infant sleeping and food‐free soothing, swaddling may reduce weight gain (Lavner et al., 2019).

The framework echoes sentiments of the ‘shared feeding responsibility’ paradigm discussed above in which both parents and infants play an equally important role. Discouraging the use of feeding as the first parental response to infant distress, swaddling was promoted as one of a handful of alternative soothing strategies alongside side‐stomach sleep position, shushing, swinging, and (non‐nutritive) suckling (Karp, 2002). Previous randomized controlled trials of this program have demonstrated fewer nocturnal and daily feeds from age 3 to 16 weeks compared to control group infants and slower weight gain over the first year of life, particularly in breastfed infants (Anzman‐Frasca et al., 2013; Paul et al., 2014; Paul et al., 2018; Savage et al., 2016).

In several of the above‐reviewed studies swaddling is positioned as a novel weight regulation tool. Could swaddling be the ‘drug’ that combats the obesity epidemic, akin to metabolism regulating pills? Is it ‘dose‐specific’? Despite its success at reducing feed frequency in breastfed infants, arguably swaddling could *promote* obesity in bottle‐fed infants where intake is governed by milk flow. During feeds, infants use instinctive body language to indicate when they have reached satiation. Arching of the back, pushing away, open arms at side of body, and open or relaxed fingers, are all cues that the infant is ready to stop feeding (White & Bryan, 2002). If infants are swaddled during feeds, these behaviors are masked and even disabled, providing the opportunity for overfeeding. Overfeeding risk is of particular concern within Western settings, where obesity is prominent and where obesogenic practices such as infant feeding with artificial breast milk substitutes, are the norm (Tomori et al., 2016). We therefore maintain that such recommendations are not good reasons for swaddling infants, regardless of feeding method, whether breastfed, formula fed or bottle fed with expressed breastmilk.

## 2 CONCLUSION

Whether swaddling is beneficial for some babies, for instance those that are premature and tube‐fed, remains unknown. However, for healthy full‐term infants, the potential for swaddling to undermine breastfeeding is a cause for concern. Few studies have examined the direct relationship between swaddling and breastfeeding; those that have overlook important contextual variables necessary to draw firm conclusions. There is insufficient evidence from randomized trials or observational studies to inform robust recommendations. However, any recommendations about swaddling should take into account its potential to undermine breastfeeding given its role in impeding skin‐to‐skin contact, separating mother and infant bodies, supressing and obscuring early infant feeding cues, preventing optimal feeding positioning and active infant engagement in feeding, interfering with responsive feeding and shared feeding responsibility, reducing feed frequency and thereby under‐stimulating milk production. This review highlights the urgent need for studies directly examining the impact of swaddling on the above components of breastfeeding behavior and lactation. It also highlights that there is potential cause for concern in promoting swaddling as an obesity prevention mechanism for both breastfed and formula‐fed infants and that the role of infant body movements in signaling satiety should be more carefully examined.

## CONFLICT OF INTEREST STATEMENT

The authors have no conflicts of interest to disclose.

## CONTRIBUTOR STATEMENT

Allison Dixley conceptualized study and wrote the initial draft. Helen L. Ball supervised the writing process. Both authors contributed to, read and approved the final manuscript.

## FUNDING INFORMATION

This study was funded by an Economic and Social Research Council Doctoral Studentship to the first author (Grant Ref: ES/J500082/1).

## Data Availability

Data sharing is not applicable to this article as no new data were created or analyzed in this study.
